# Study on the Effect of NTO on the Performance of HMX-Based Aluminized Cast-PBX

**DOI:** 10.3390/ma15144808

**Published:** 2022-07-09

**Authors:** Pengsong Nie, Shaohua Jin, Xinyu Kou, Lixiaosong Du, Lijie Li, Kun Chen, Yu Chen, Junfeng Wang

**Affiliations:** School of Materials Science and Engineering, Beijing Institute of Technology, Zhongguancun South Street 5, Beijing 100081, China; nie920902@163.com (P.N.); jinshaohua@bit.edu.cn (S.J.); kxy8328238@163.com (X.K.); dulixiaosong@163.com (L.D.); lilijie2003@bit.edu.cn (L.L.); kchen@bit.edu.cn (K.C.)

**Keywords:** HMX, NTO, cast-PBX, thermal safety, gurney energy, numerical simulation

## Abstract

3-Nitro−1,2,4-triazol−5-one (NTO) is an explosive with broad application prospects. To study the effect of NTO content on the properties of HMX-based cast-PBX (polymer bonded explosive), five different HMX/NTO-based cast-PBXs were prepared and characterized by experiments and simulations. The results show that the addition of NTO is beneficial to reduce the mechanical sensitivity of cast-PBX, but will reduce the energy level of cast-PBX. We then found that with the increase in NTO content, cast-PBX showed a trend of first increasing and then decreasing in terms of mechanical properties, specific heat capacity (Cp) and thermal conductivity (λ). In addition, we found that the Gurney energy (*Eg*) of N30 is 2.31 kJ/g. Finally, the increase in NTO content greatly improves the thermal safety performance of the cast-PBXs, and numerical simulation of slow cook-off can be used as one reliable method to obtain the ignition location, ignition temperature and the transient temperature distribution.

## 1. Introduction

Cast-polymer bonded explosive (PBX) is a mixed explosive with high-energy elementary explosives, polymer binders, curing agents and plasticizers. It is widely used in penetrating warheads, ultra-high-speed missiles and underwater warheads [1,2,3,4]. In order to ensure the safety of weapons and ammunition in the complex battlefield environment, the research and application of insensitive cast-PBX has become pertinent.

3-Nitro−1,2,4-triazol−5-one (NTO) is an explosive with broad application prospects. The detonation velocity of NTO is higher than trinitrotoluene (TNT), triamino trinitrobenzene (TATB), and its sensitivity lower than cyclotrimethylene trinitramine (RDX) and octahydro−1,3,5,7-tetranitro−1,3,5,7-tetrazocine (HMX). It has excellent comprehensive performance, making it the preferred material for the preparation of highly energy-insensitive PBX [5,6]. Highly energy-insensitive cast-PBX containing NTO has been widely used in insensitive ammunition [7,8,9].

In order to realize the insensitiveness of cast-PBX, a series of NTO-based cast-PBXs for large shells have been studied in France [10], such as B2267A (RDX/NTO/hydroxyl-terminated polybutadiene (HTPB)) and B2268A (RDX/NTO/AI/HTPB). These two formulas have low sensitivity and excellent mechanical properties. The densities of these are 1.65 g/cm^3^ and 1.76 g/cm^3^, respectively, with detonation velocities of 7680 m/s and 7440 m/s, respectively, and a critical initiation diameter of 30–36 mm. The PBXW series of NTO-based cast-PBXs have been launched in the USA [11], among which PBXW−126 has good detonation performance and low sensitivity. After that, ATK company [5] developed a cast-PBX DLE-C054 (NTO/RDX/HTPB), which fully met the demand for insensitive ammunition, and could be mass-produced. Tao Chai et al. [12] applied the supercritical carbon dioxide coating method to study the preparation process of HMX/NTO-based PBX, and found that supercritical fluid coating technology is a pollution-free and effective way to prepare NTO-based water-soluble explosives. Nana Wu et al. [13] used prepared NTO/HMX-based explosive. The density was 1.79 g/cm, and the detonation velocity was 8200 m/s. The formula has excellent molding and detonation performance as well as low sensitivity. Although the effect of NTO on the thermal safety performance of compress-PBX has been reported [14], the effect of NTO on the performance of aluminized cast-PBX has never been reported.

This paper takes 32% octahydro−1,3,5,7-tetranitro−1,3,5,7-tetrazocine (HMX) and NTO as the main body with which to carry out the basic application research of highly energy-insensitive cast-PBXs. HMX/NTO-based aluminized cast-PBXs with different NTO and HMX ratios were prepared, and the effects of NTO and HMX ratios on the basic properties of cast-PBXs such as mechanical sensitivity, mechanical properties, detonation performance, and thermal safety were explored. Finite element numerical simulations were used to obtain the internal temperature distribution of the explosive during slow cook-off tests.

## 2. Experiment

### 2.1. Preparation of Cast-PBXs

In this paper, five kinds of HMX/NTO-based aluminized cast-PBX samples (Table 1) were prepared by the mixing-casting-curing process. The specific preparation steps are as follows:

(1) Each component is dried before the experiment at a drying temperature of 65 °C. Their water content should be controlled at about 2%.
(2) According to the process requirements, all the weighed components are poured into a 5 L vertical kneading machine, so that different components are evenly mixed under vacuum (0.05 MPa) and heating conditions (60 °C).
(3) The material is quickly poured into the vacuum cast equipment, and the material is poured into the prepared mold (Φ 20 × 20 mm, Φ 20 × 30 mm, Φ 30 × 30 mm, Φ 40 × 40 mm, Φ 50 × 20 mm) at a speed of about 5 mL/min under heat preservation (60 °C) and vacuum conditions (0.05 MPa).
(4) The mold with the cast explosives was heated and cured for 7 d at 60 °C. After curing, the molds were cooled to room temperature, and the explosives were released from the molds.

### 2.2. Characterization

The microscopic morphologies of different cast-PBXs were characterized by the MIRA3 XM scanning electron microscope (SEM), produced by the Tescan Company of the Czech Republic. The test voltage was set at 3.5 kV, and the samples were treated with gold spraying before undergoing the test.

### 2.3. Mechanical Sensitivity

The impact sensitivity and friction sensitivity of different cast-PBXs were characterized by WL−1-type drop weight tester and WM−1-type friction sensitivity tester. The test conditions of impact sensitivity are set as follows: the explosive quantity is (20 ± 1) mg; the weight of the drop ball is (10.00 ± 0.01) kg; the drop height is (250 ± 1) mm; the test is carried out at room temperature. The test conditions of friction sensitivity are set as follows: the explosive quantity is (20 ± 1) mg; the swing angle is (90 ± 10) °; the gauge pressure is (3.92 ± 0.07) MPa; the test is carried out at room temperature; the relative humidity does not exceed 80%.

### 2.4. Mechanical Properties

Tensile strength: the tensile strength of different cast-PBXs samples was characterized according to the “Brazilian test method”. The radial pressure was applied to the explosive sample with size of Φ 20 × 20 mm at a loading rate of 0.5 mm/min.

Compressive strength: the compressive strength of different cast-PBXs samples was characterized according to GJB772A−97 method 416.1. In this method, the tested sample is placed between the upper and lower pressing plates of the material testing machine, and the quasi-static axial compression load is applied until the pattern is broken. The maximum load that can be sustained on the unit cross-sectional area is the compressive strength. Test conditions: an axial load is applied to the explosive sample with size of Φ 20 × 20 mm at a loading rate of 0.5 mm/min until the sample is destroyed or the testing machine reaches the maximum load.

Shear strength: the shear strength of different cast-PBXs samples was characterized according to GJB772A−97 method 415.1. In this method, the tested sample is placed in the shear fixture of the material testing machine, and the uniaxial static load is applied to make the sample fracture and break under the shear force. The shear stress at the fracture is calculated as the shear strength. Test conditions: the explosive sample with size of Φ 20 × 30 mm was placed in a shearing fixture, and the loading rate was 10 mm/min.

### 2.5. Detonation Properties

**Figure 1:**
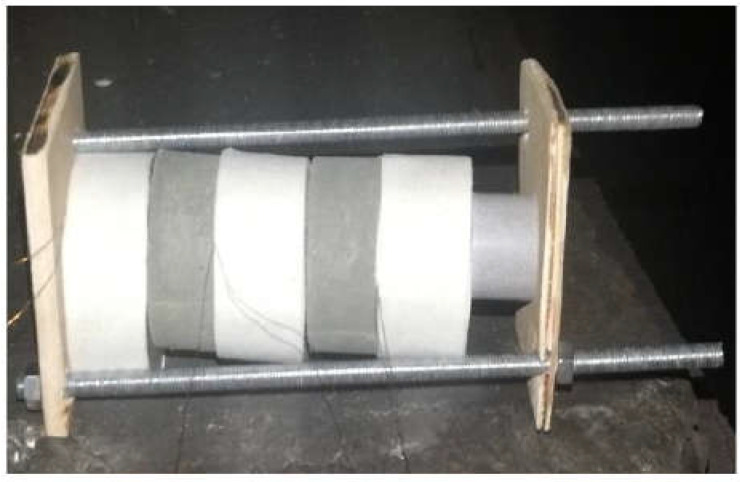
The diagram of detonation velocity test.

The detonation velocity of different cast-PBXs was characterized according to GJB 772A−97 method 702.1, the “Electrical Test Method”. In this method, the propagation time of detonation wave in a certain length of explosive column is measured by means of time meter and electric probe, and the detonation velocity of the sample is calculated. The size of the explosive sample is Φ 40 × 40 mm. The diagram of the detonation velocity test was shown in Figure 1.

The detonation heat of different cast-PBXs was characterized according to GJB772A−97 method 701.1. In this method, the explosive sample is detonated under vacuum conditions, and distilled water is used as the temperature measuring medium to monitor the rise of water temperature, and the explosion heat of the sample per unit mass is calculated according to the heat capacity and temperature rise. Test conditions: the size of explosive sample is Φ 30 × 30 mm and the laboratory temperature is controlled at 15~30 °C.

### 2.6. Thermal Characteristic

The specific heat capacity (Cp) of different cast-PBXs was characterized by DSC 200F3, produced by NETZSCH, Germany. Test conditions: there is an N_2_ atmosphere; the sample mass is 12.960 mg, the temperature range is 0~50 °C; the heating rate is 10 °C·min^−1^.

The thermal conductivity (λ) of different cast-PBXs was characterized by TCI type thermal conductivity measuring apparatus produced by Canadian C-THERM company. The test temperature is set to 25 °C.

### 2.7. Thermal Safety Performance

The adiabatic decomposition performance of different cast-PBXs was investigated by accelerating rate calorimeter (ARC). In total, 200 mg samples of different cast-PBXs were placed in a hastelloy vessel with a mass of 21.1 g, and were tested by the “heat-wait-search” procedure with temperature increments of 5 °C. The starting temperature was 50 °C and the ending temperature was 260 °C (self-heating rate = 0.02 °C·min^−1^).

**Figure 2:**
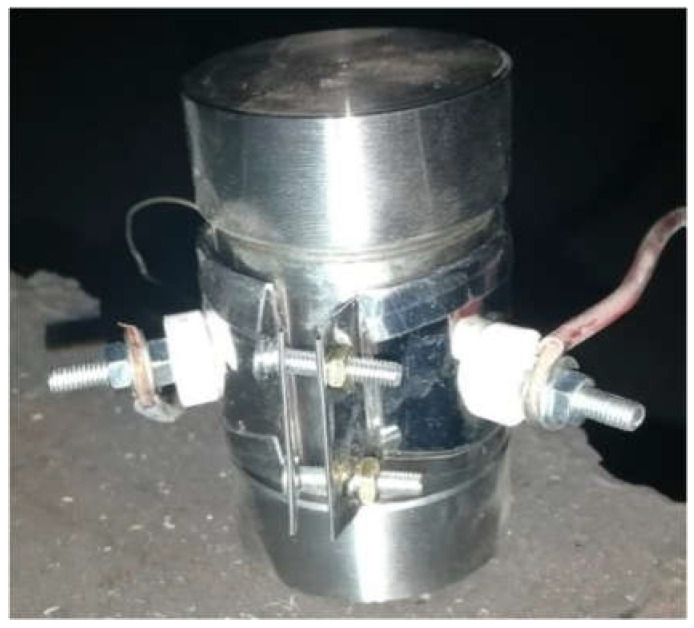
The assembly diagram for slow cook-off test.

The slow cook-off tests of different cast-PBXs were conducted to evaluate the thermal safety performance of the munition. Test conditions: the shell material of the slow cook-off bomb is 45^#^ steel; the inner diameter is Φ 40 mm and the thickness of the shell is 3 mm; the heating rate is 1 °C/min. After the test, the slow cook-off performance of different cast-PBXs was evaluated by the damage to the bomb shell after the tests. The assembly diagram for the slow cook-off test is shown in Figure 2.

### 2.8. Cylinder Test

**Figure 3:**
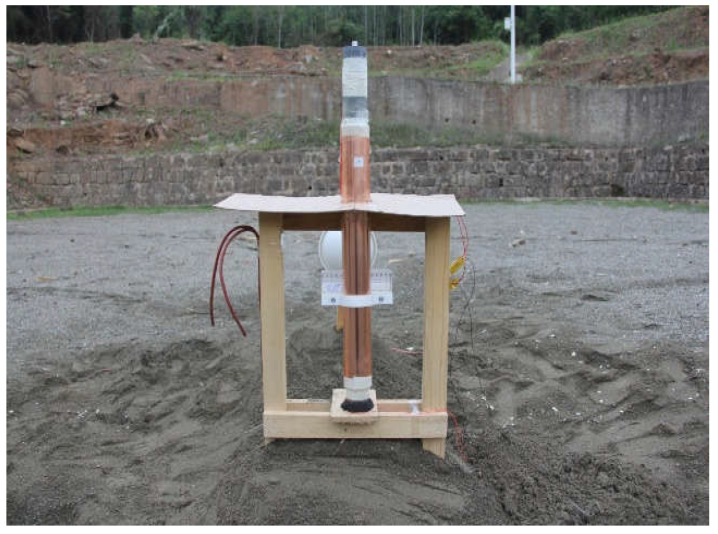
The cylinder test device.

To obtain the metal-driven ability of NTO/HMX-based cast-PBX, a 50 mm cylinder test was carried out for N30. Owing to the cost of the cylinder test, only N30 with better comprehensive performance was selected as the test object. The density of N30 explosive is 1.706 g·cm^−3^. The cylinder was made of oxygen-free copper, with a length of495 mm. The dimensions of the copper tube is 50 mm.The expansion process of the cylinder wall at a distance of 295 mm from the detonation surface was recorded by the high-speed scanning camera. The cylinder test device is shown in Figure 3.

The Gurney energy (*E_g_*) reflects the ability of explosive to drive the metal. The *E_g_* can be obtained by:

(1) $$E_{g}=\frac{1}{2}\left(\beta +\frac{1}{2}\right)v^{2}$$

Note: v: the maximum expansion velocity of cylinder wall; β: mass ratio per unit length of cylinder and explosive.

### 2.9. Numerical Simulation of Slow Cook-Off

**Figure 4:**
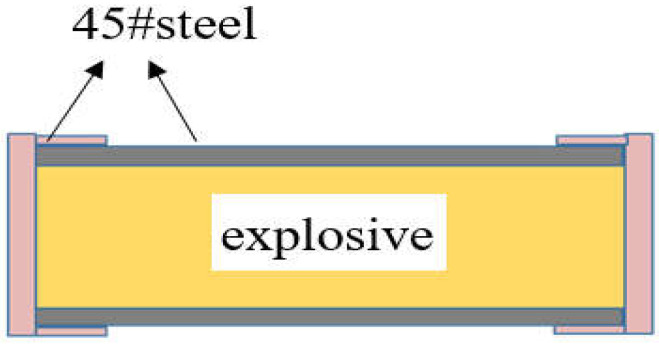
The simulation diagram of slow cook-off bomb.

According to the parameters of the test, the process of a slow cook-off was simulated by Fluent to obtain the ignition location, ignition temperature and the transient temperature distribution, which was not examined by the test. In the simulation, the shell material of the slow cook-off bomb is 45# steel. The inner diameter is Φ 40 mm and the thickness of the shell is 3 mm. The outer wall of the slow cook-off bomb is set as the heating surface; the heating rate and the self-heating source term S of cast-PBXs are loaded through the UDF interface of Fluent; the slow cook-off bomb is heated at a heating rate of 1 °C/min until the cast-PBXs ignites. The simulation diagram of slow cook-off bomb is shown in Figure 4.

## 3. Results and Discussion

### 3.1. Microscopic Morphology

**Figure 5:**
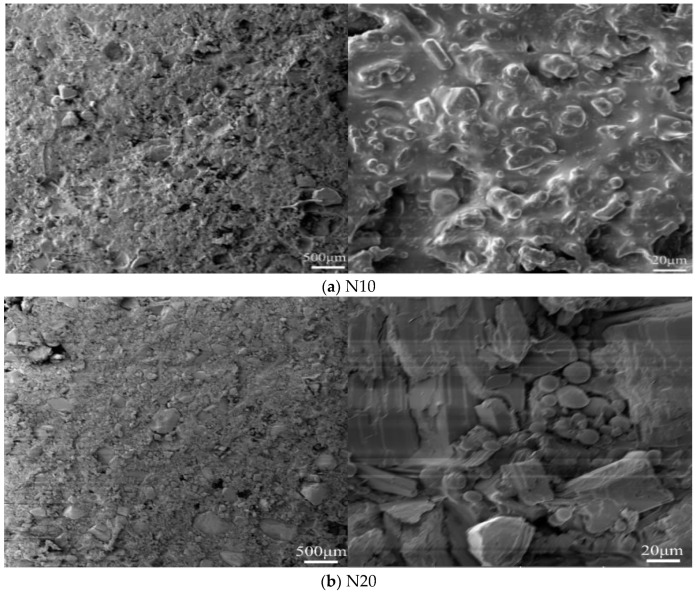

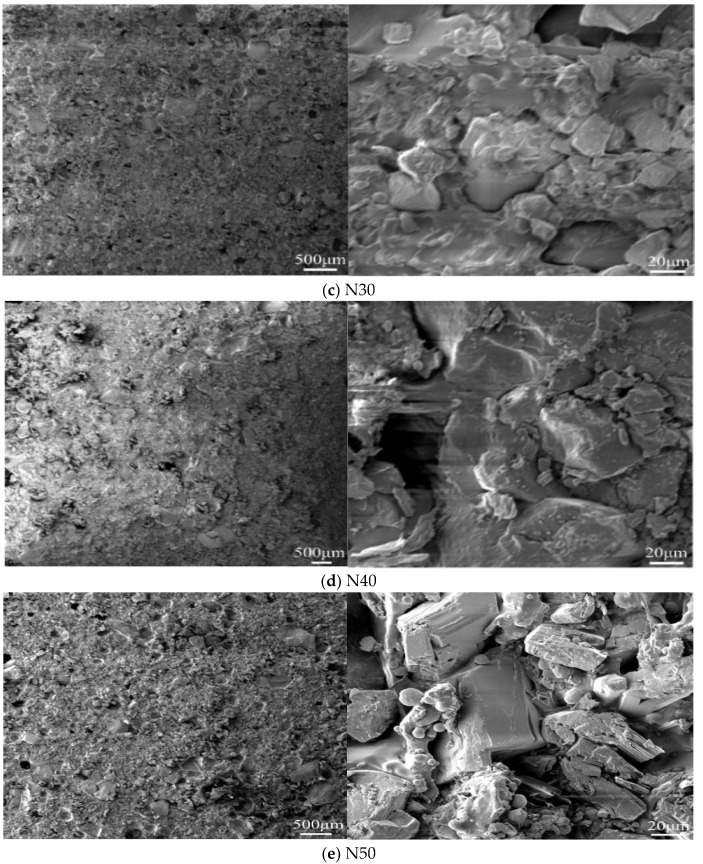
The SEM photographs.

The microscopic morphology of five kinds of cast-PBXs were characterized by SEM. The results are shown in Figure 5.

Through Figure 5, we can observe the debonding of crystal grain caused by mechanical processing, as well as some defects of the crystal grain surface caused by crystal accumulation. In terms of crystal bareness and uniformity of binder distribution, N10 and N20 are better than N30, N40 and N50. With the increase in NTO content, the degree of irregularity of the binder system increases, as do the number of exposed explosive crystals and aluminum powder particles.

### 3.2. Mechanical Sensitivity

The measurement results of mechanical sensitivity for five kinds of HMX/NTO-based aluminized cast-PBXs are listed in Table 2. GOL−42 [15] is the HMX-based aluminized cast-PBX without NTO (75%HMX,15%Al,10%binder)

In Table 2, the impact sensitivity and friction sensitivity of the five HMX/NTO-based aluminized cast-PBXs are both below 25%. With the increase in the NTO content, the impact sensitivity and friction sensitivity decreased. When the NTO content was changed from 10% to 20%, the impact sensitivity fell from 24% to 10%. In this range, the addition of NTO significantly reduced the mechanical sensitivity of NTO/HMX-based aluminized cast-PBXs. Because of the addition of a compound sensitivity reduction reagent, the impact sensitivity of GOL−42 is only 4%, but friction sensitivity of GOL−42 is still quite high. The sensitivity of an energetic material is largely determined by its own structure. For NTO, the lone pair of electrons on the nitrogen atom joins the conjugation, which leads to enhanced aromaticity of the ring, thereby improving the thermal stability. The melting point of NTO also rises above its decomposition temperature due to the presence of intermolecular hydrogen bonds. Additionally, NTO is less sensitive to the mechanical effects of impact and friction, which prevents the rapid generation of hot spots. The HMX molecule is an eight-membered heterocyclic structure with alternating carbon and nitrogen, which is more sensitive to the mechanical effects of impact and friction. Therefore, the higher the NTO content, the lower the probability of generating hot spots when subjected to mechanical action.

### 3.3. Mechanical Properties

**Figure 6:**
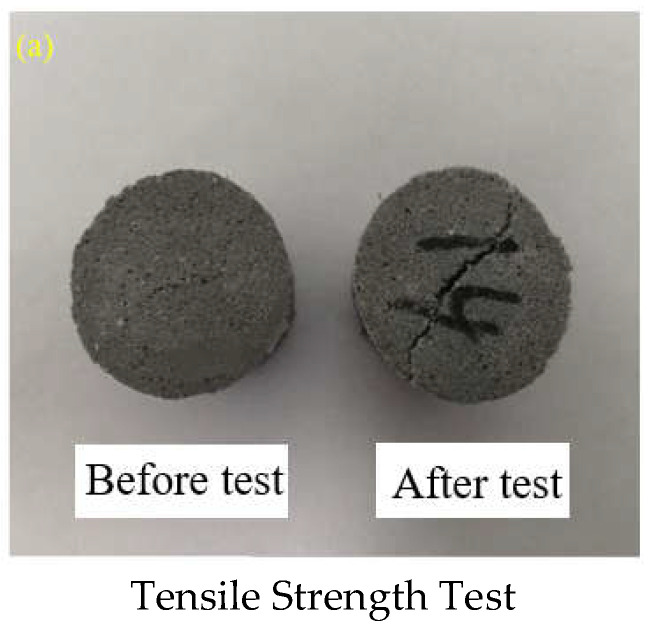

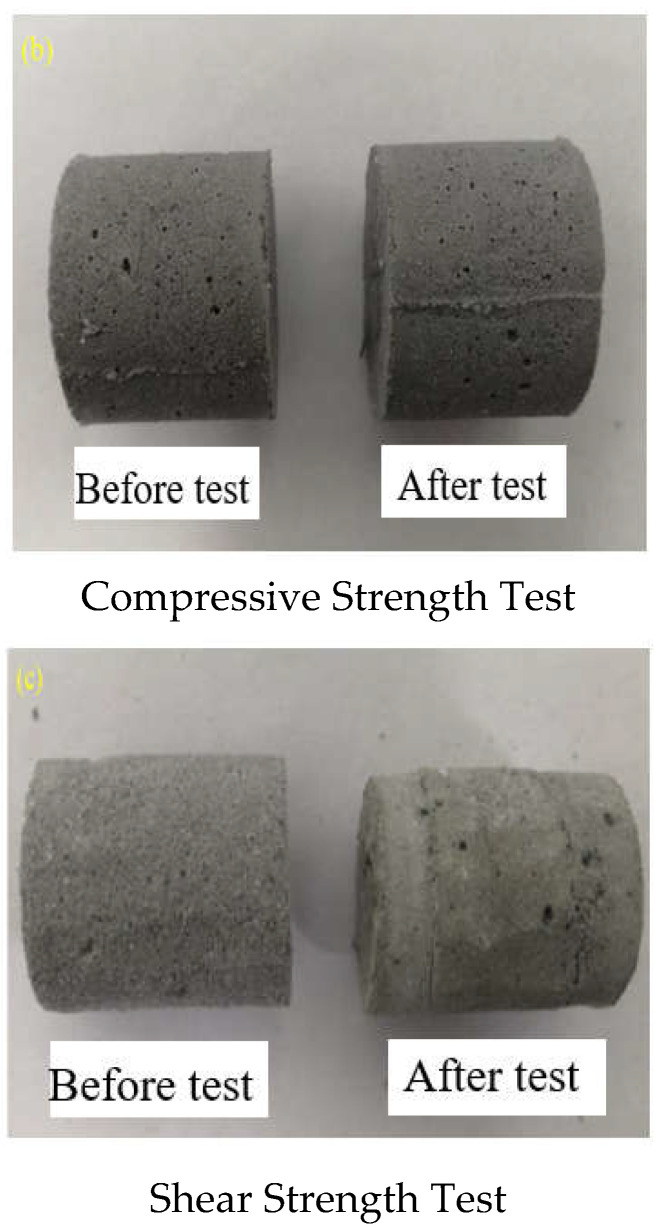
The change of N30 appearance after tests.

After curing and demolding, the Φ 20 × 20 mm grains were used for tensile strength and compressive strength tests and the Φ 20 × 30 mm is used for shear strength tests. In order to make the test results more accurate, 5 grains were selected for each test, and the results given were the average of the five grains. Taking N30 as an example, Figure 6 shows the change of N30 appearance after tests.

It can be found from Figure 6 and Table 3 that the three mechanical property parameters all showed a trend of first increasing and then decreasing with the increase in NTO content. N30 has the best rigidity, and is less likely to deform after being stimulated by mechanics. Compared with GOL−42, the addition of NTO reduces the mechanical properties of HMX-based aluminized cast-PBX.

### 3.4. Detonation Properties

The detonation speed is the basic parameter to measure the energy level of explosives. The detonation velocity results and the detonation heat results of cast-PBXs are shown in Table 4.

It can be found from Table 4 that the measured detonation velocity of each cast-PBXs is sorted in order: N10 > N30 > N20 > N40 > N50. With the increase in NTO content in the cast-PBX, the measured detonation velocity showed a decreasing trend.

The changing trend of detonation heat of cast-PBXs is consistent with detonation velocity. The HMX molecule has higher nitrogen content and more initiating bonds, so it has higher detonation heat than NTO. Therefore, with the increase in HMX content, the detonation heat tends to increase.

### 3.5. Thermal Characteristic

**Figure 7:**
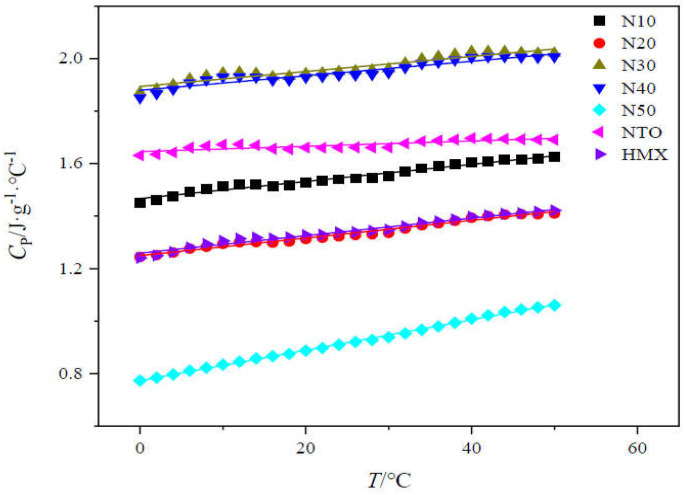
C_P_-T curves of five NTO/HMX-based cast-PBXs, NTO and HMX.

The specific heat capacity-temperature curves (C_P_−T) of five different HMX/NTO-based cast-PBXs (temperature range of 0~50 °C) are shown in Figure 7. The specific heat capacity (C_P_) at 25 °C and thermal conductivity(λ) of different cast-PBXs are listed in Table 5.

From the data in Table 5, it can be found that at 25 °C, the C_P_ of N30 and N40 is larger, and the C_P_ of N10 and N50 is smaller. With the increase in NTO, the C_P_ and λ of the cast-PBXs showed a trend of first increasing and then decreasing. Among the cast-PBXs, N20 has the highest λ. Large C_P_ means the explosive has a better ability to absorb heat and small λ means better thermal insulation of explosives. When the explosive with large C_P_ and small λ is stimulated to form a hot spot, its heat will be absorbed, resulting in an explosion from self-accelerating thermal decomposition reaction being prevented. The addition of NTO can greatly reduce the thermal conductivity of explosives. From the data in Table 2 and Table 5 it can be determined that although N50 has the smallest C_P_, its value of λ is only 0.46. This indicates that it has the best thermal insulation effect and can prevent the formation of hot spots when externally stimulated, meaning the mechanical sensitivity of N50 is also the lowest.

### 3.6. Thermal Safety Performance

**Figure 8:**
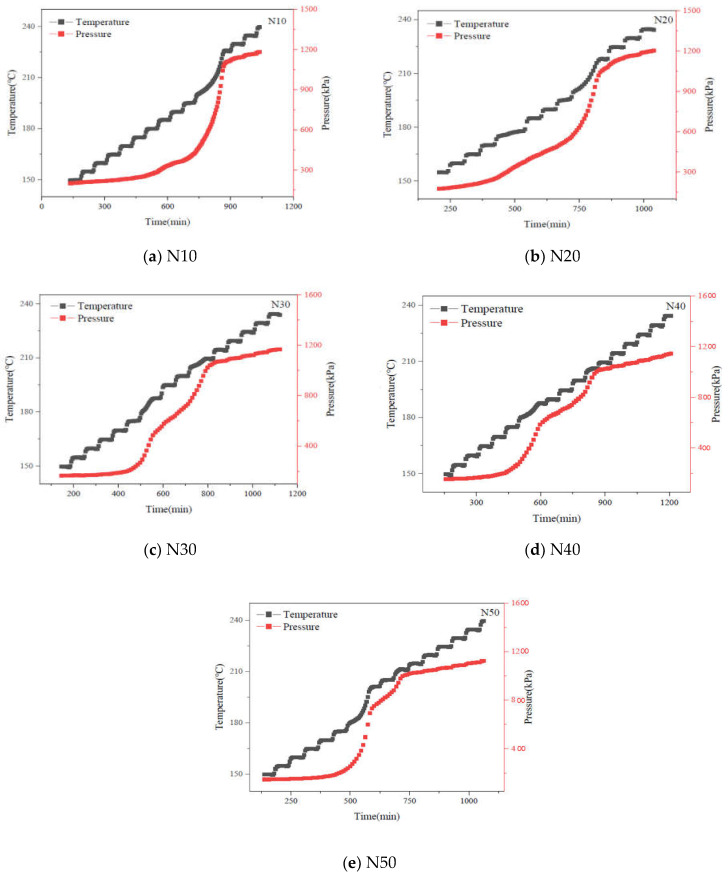
The curves of pressure and temperature with time.

The adiabatic thermal decomposition behavior of HMX/NTO-based aluminized cast-PBXs were investigated by ARC. The variation of pressure and temperature with time during the whole process were shown in Figure 8.

Combining the factors of pressure and temperature, different cast-PBXs have two adiabatic thermal decomposition stages. The adiabatic thermal decomposition reactions of N10 and N20 mainly occur in the second stage, while N30, N40, N50 mainly occur in the first stage. With the increase in the relative content of NTO, the slow decomposition stage was prolonged, resulting in more reactants being consumed in this stage, as well as more gas being produced in the reaction. In a confined space, if the gas generated in the first stage is enough to rupture the shell, the explosive will be in an unsealed state. As such, the reaction will not proceed to the second stage, and the detonation reaction will not occur easily. It can be carried out at night to explain the phenomenon that the lower the temperature of the slow cook-off experiment, the easier it is to pass.

**Figure 9:**
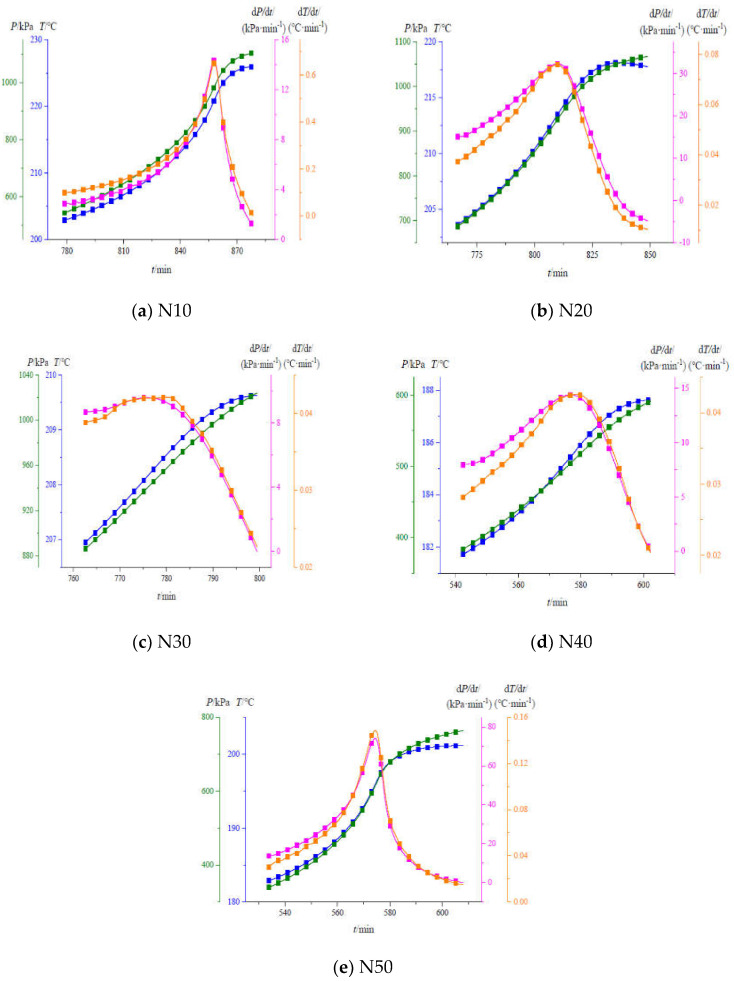
T, P, dT/dt, dP/dt curves with time.

Figure 9 shows that temperature (T), pressure (P), rate of temperature rise (dT/dt) and rate of pressure change (dP/dt) curves with time during thermal decomposition of N10, N20, N30, N40 and N50. Table 6 shows adiabatic thermal decomposition characteristic parameters of five different HMX/NTO-based aluminized cast-PBXs.

Figure 9 and Table 6 show that the initial decomposition temperature of N10, N20, N30, N40 and N50 are in the order of N40 < N50 < N10 < N20 < N30. To a certain degree, the initial decomposition temperature reflects the difficulty of conducting adiabatic thermal decomposition reactions. In the main stage of adiabatic decomposition, the reaction is more difficult to create due to the higher initial decomposition temperature of N30. During the adiabatic decomposition reaction, the dT/dt and dP/dt of the five cast-PBXs increased first and then decreased. With the increase in NTO content, ΔT_ad_, *β_m_*, *t_m_*, P_m_, and Q of different cast-PBXs showed a trend of first decreasing and then increasing. In summary, when the NTO and HMX contents are closer in value, the reaction of HMX/NTO-based aluminized cast-PBXs in the main stage of adiabatic thermal decomposition is more gradual, and the possibility of thermal explosion is reduced.

**Figure 10:**
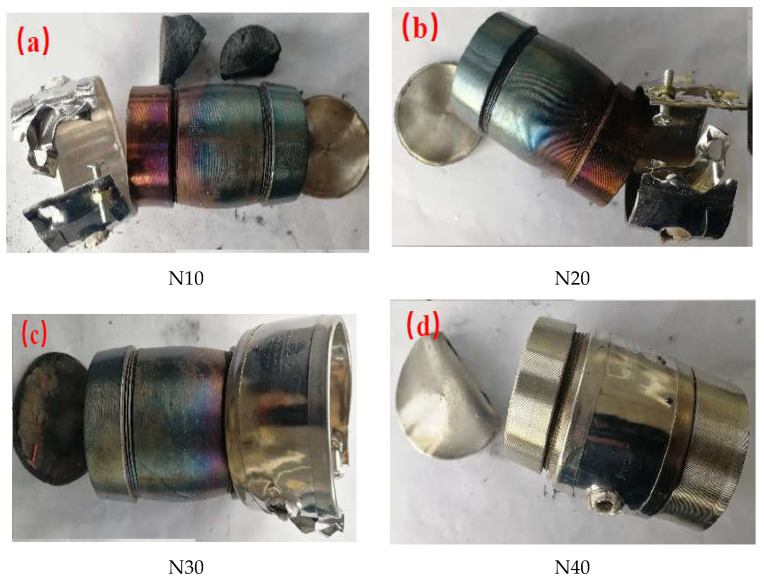

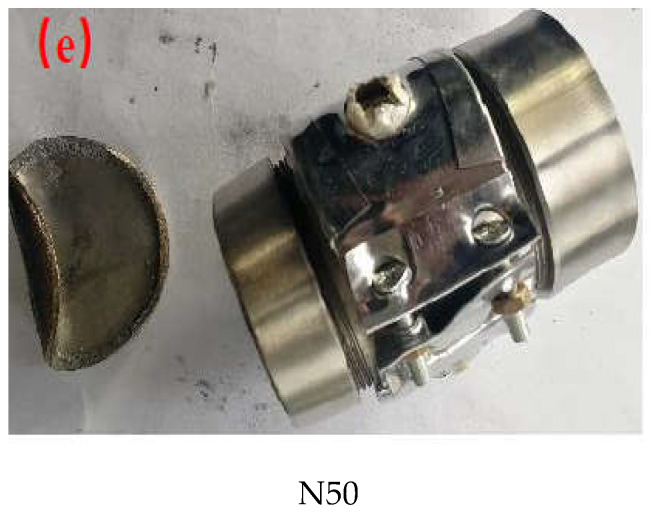
The degree of shell breakage after slow cook-off test.

The slow cook-off test results of five cast-PBXs are listed in Table 7. Figure 10 shows the degree of shell breakage after slow cook-off test.

Combined, Figure 10 and Table 7 show five different cast-PBXs’ slow cook-off bombs to have one separated end cap. The shells of N10, N20, and N30 have obvious deformation, while the shells of N40 and N50 are intact, with only slight deformation. It can be considered that the N10, N20, and N30 have deflagration reactions, while N40 and N50 have combustion reactions. Owing to the addition of the compound sensitivity reduction reagent, the response type of GOL−42 is combustion.

According to the results of the slow cook-off test, the response temperatures of different cast-PBXs are all above 210 °C. With the increase in NTO content in the cast-PBX, the response temperature shows a trend of first increasing and then decreasing, which may be due to the thermal decomposition temperature of HMX and NTO being relatively close. With the increase in NTO content, the response type of slow cook-off gradually changed from deflagration to combustion, indicating that the addition of NTO is beneficial to passing the slow cook-off test.

### 3.7. Cylinder Test

**Figure 11:**
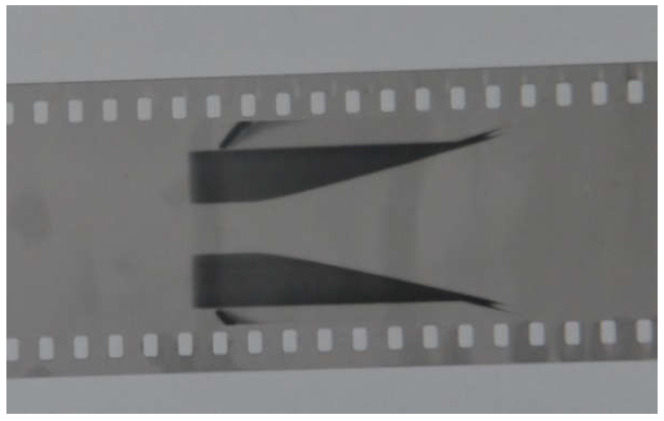
Photograph of expansion process for the cylinder wall.

The scanning result of the expansion process of the cylinder wall at the slit position is shown in Figure 11.

**Figure 12:**
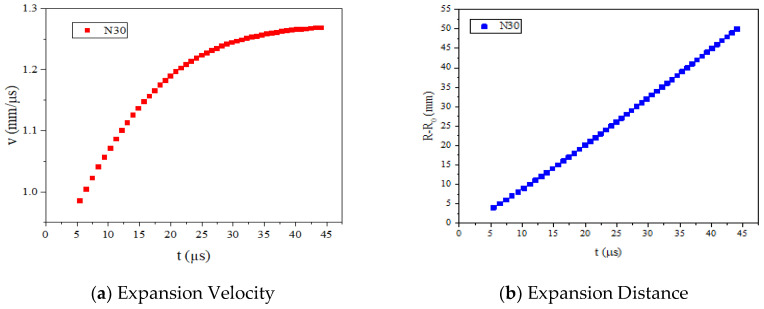
Expansion velocity and expansion distance curves.

The v-t and (R-R_0_)-t curves are shown in Figure 12. (v: the expansion velocity of cylinder wall; R-R_0_: the expansion distance of cylinder wall; t: time)

From Figure 12, we can see that the maximum expansion velocity of the cylinder walls is 1.268 mm/μs. According to Formula (1), we can obtain that the E_g_ of N30 is 2.31 kJ/g.

### 3.8. Numerical Simulation of Slow Cook-Off

**Figure 13:**
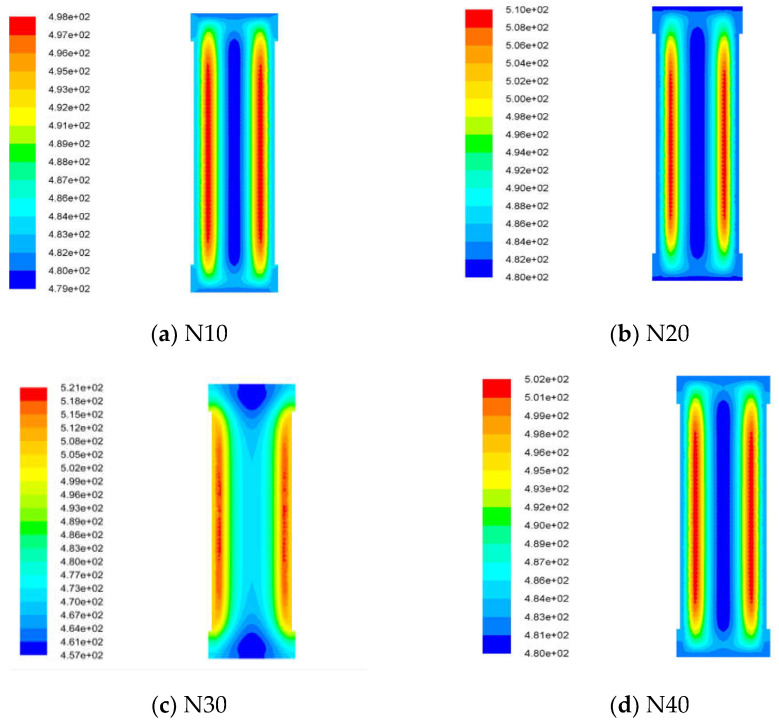

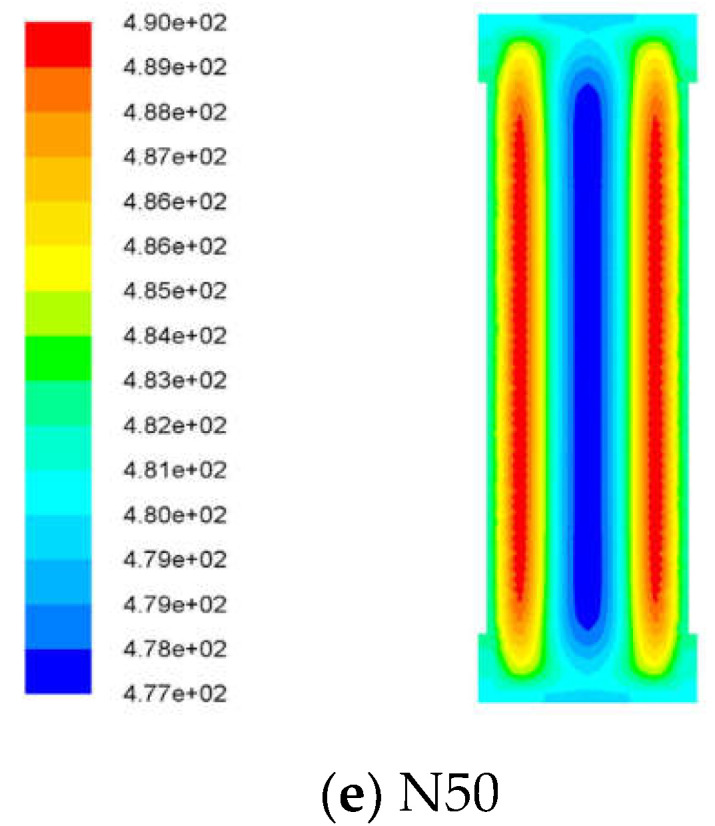
The ignition condition of different cast-PBXs.

The ignition location, ignition temperature and the transient temperature distribution of different cast-PBXs were shown in Figure 13.

From Figure 13, we can see that the ignition positions are on both sides and, and that the ignition temperature of different cast-PBXs were 224.85 °C, 236.85 °C, 247.85 °C, 228.85 °C, 216.85 °C, respectively. Comparing the simulated ignition temperature to the response temperature of the slow cook-off test, we find the error to be within 2%. This illustrates the accuracy of the slow cook-off numerical model. The simulated ignition model is not only a good supplement to the slow cook-off test, but also helps us predict the response temperature of the slow cook-off test in the future.

## 4. Conclusions

Five kinds of HMX/NTO-based aluminized cast-PBX samples were prepared and characterized by experiments and numerical simulation. This paper mainly focuses on the effect of HMX/NTO ratio to the performance of cast-PBXs. The main conclusions were as follows:

(1) With the increase in NTO content in cast-PBXs, the mechanical sensitivity, detonation velocity and the detonation heat of cast-PBXs decreased. This shows that the addition of NTO will enhance the safety of cast-PBX but will lead to a decrease in energy.
(2) For mechanical properties, specific heat capacity (Cp) and the thermal conductivity (λ) there was a trend of initially increasing and then decreasing with the increase in NTO content. Among the cast-PBXs, N30 has the best rigidity and highest Cp. Through the cylinder test, the Gurney energy (*E_g_*) of N30 is 2.31 kJ/g.
(3) From the results of ARC tests and slow cook-off tests, we can know that the addition of NTO greatly improves the thermal safety of the cast-PBX and reduces the probability of cast-PBX detonating under thermal stimulation. The error of simulated ignition temperature and response temperature to the slow cook-off test is kept within 2%. Numerical simulation makes up for the deficiency of the experiment and can be used as a method for predicting the response temperature in the future.

## Figures and Tables

**Table 1:** The formulation of cast-PBXs.

Formulation	NTO%	HMX%	Al%	Binder%
N10	10	55	20	15
N20	20	45	20	15
N30	30	35	20	15
N40	40	25	20	15
N50	50	15	20	15

**Table 2:** The measurement results of mechanical sensitivity.

Formulation	Impact Sensitivity (P_1_%)	Friction Sensitivity (P_F_%)
N10	24	15
N20	10	8
N30	8	4
N40	5	4
N50	4	2
GOL−42 [15]	4	32

**Table 3:** The measurement results of mechanical properties.

Formulation	Compressive Strength	Tensile Strength	Shear Strength
N10	1.06	0.25	0.26
N20	1.20	0.26	0.33
N30	2.26	0.39	0.41
N40	1.46	0.35	0.45
N50	1.57	0.33	0.40
GOL−42 [15]	3.82	1.11	0.96

**Table 4:** The detonation velocity and detonation heat results.

Formulation	Detonation Velocity/(m/s)	Detonation Heat/(kj/kg)
N10	7705	6792.20
N20	7522	6564.05
N30	7598	6562.14
N40	6882	5881.03
N50	6860	5798.85
GOL−42 [15]	8251	6707

**Table 5:** The C_P_ at 25 °C and λ of different cast-PBXs.

Formulation	25 °C C_P_/J·g^−1^·°C^−1^	λ/W·m^−1^·K^−1^
N10	1.33	0.64
N20	1.55	0.85
N30	1.97	0.69
N40	1.95	0.54
N50	0.92	0.46

**Table 6:** Adiabatic thermal decomposition characteristic parameters.

Parameter	N10	N20	N30	N40	N50
T_0_/°C	202.89	203.63	206.95	181.71	182.89
T_f_/°C	225.95	218.13	209.62	187.62	201.17
ΔT_ad_/°C	23.06	14.50	2.67	5.91	18.28
*β_m_*	0.65	0.32	0.10	0.14	0.74
*t_m_*	79.12	43.06	13.23	34.32	40.51
P_m_	1112.07	1067.59	1041.69	609.70	774.59
Q	35.74	19.29	5.26	11.52	16.82

Note: T_0_, Initial decomposition temperature; T_f_, Termination decomposition temperature; ΔT_ad_, adiabatic temperature rise; *β_m_*, Maximum rate of temperature rise; *t_m_*, The time required for the system to reach the maximum rate of temperature rise; P_m_, The maximum decomposition pressure; Q, Adiabatic decomposition heat.

**Table 7:** The slow cook-off test results.

Formulation	Response Temperature/°C	Response Type
N10	225	deflagration
N20	238	deflagration
N30	251	deflagration
N40	234	combustion
N50	216	combustion
GOL−42 [15]	216	combustion
